# Challenges and Possibilities of Cell-Based Tissue-Engineered Vascular Grafts

**DOI:** 10.34133/2021/1532103

**Published:** 2021-02-18

**Authors:** Junichi Saito, Makoto Kaneko, Yoshihiro Ishikawa, Utako Yokoyama

**Affiliations:** ^1^Department of Physiology, Tokyo Medical University, Shinjuku-ku, Tokyo, Japan; ^2^Cardiovascular Research Institute, Yokohama City University, Yokohama, Kanagawa, Japan; ^3^Faculty of Science and Technology, Meijo University, Nagoya, Aichi, Japan

## Abstract

There is urgent demand for biologically compatible vascular grafts for both adult and pediatric patients. The utility of conventional nonbiodegradable materials is limited because of their thrombogenicity and inability to grow, while autologous vascular grafts involve considerable disadvantages, including the invasive procedures required to obtain these healthy vessels from patients and insufficient availability in patients with systemic atherosclerosis. All of these issues could be overcome by tissue-engineered vascular grafts (TEVGs). A large body of evidence has recently emerged in support of TEVG technologies, introducing diverse cell sources (e.g., somatic cells and stem cells) and novel fabrication methods (e.g., scaffold-guided and self-assembled approaches). Before TEVG can be applied in a clinical setting, however, several aspects of the technology must be improved, such as the feasibility of obtaining cells, their biocompatibility and mechanical properties, and the time needed for fabrication, while the safety of supplemented materials, the patency and nonthrombogenicity of TEVGs, their growth potential, and the long-term influence of implanted TEVGs in the body must be assessed. Although recent advances in TEVG fabrication have yielded promising results, more research is needed to achieve the most feasible methods for generating optimal TEVGs. This article reviews multiple aspects of TEVG fabrication, including mechanical requirements, extracellular matrix components, cell sources, and tissue engineering approaches. The potential of periodic hydrostatic pressurization in the production of scaffold-free TEVGs with optimal elasticity and stiffness is also discussed. In the future, the integration of multiple technologies is expected to enable improved TEVG performance.

## 1. Introduction

Coronary artery disease, the leading cause of adult death in developed countries [1], is treated by coronary angioplasty and coronary artery bypass grafting. Graft surgeries currently utilize autologous blood vessels (e.g., the saphenous vein and the internal thoracic artery) because small-diameter artificial grafts are not feasible due to their thrombogenicity [2]. Yet autografts involve considerable disadvantages including the invasive procedures required to obtain them and insufficient graft availability, especially in patients with atherosclerosis. To circumvent these difficulties, small-diameter vascular grafts are urgently needed, not only for patients with coronary artery disease but also for patients with chronic kidney diseases requiring hemodialysis. Patients who are not candidates for arteriovenous fistula can receive arteriovenous graft surgery using nonautologous vascular grafts, such as expanded polytetrafluoroethylene (ePTFE) grafts [3], but the median patency time of arteriovenous grafts was found to be only 10 months because of frequent thrombosis and infection [4]. Pediatric patients also need improved vascular grafts. A major cause of neonatal death is congenital heart disease, which affects approximately 1% of living newborns [5]. Congenital heart diseases are characterized by defects or malformations of one or more cardiovascular structures; their treatment sometimes requires implantation of exogenous materials during the neonatal and/or early infant periods. As artificial materials have no growth potential, these patients require frequent subsequent operations throughout their development due to recurring relative stenosis [6]. Thus, biologically compatible vascular grafts are urgently required for both adult and pediatric patients.

Tissue engineering involves the combination of engineering technology and medical science toward generating functional biological constructs [7]. In 1986, Weinberg and Bell pioneered cell-seeded tissue-engineered vascular grafts (TEVGs) [8] containing three different cell types, endothelial cells (ECs), smooth muscle cells (SMCs), and fibroblasts, which mimicked the tunica intima, media, and adventitia, respectively. These vascular mimics had extremely poor mechanical properties (burst pressure < 10 mmHg) even with the addition of a polyethylene terephthalate mesh for support (burst pressure: 120-180 mmHg). The necessity of adding an artificial material like polyethylene terephthalate also deprived the grafts of any growth or remodeling potential. Even though their engineered grafts were never implanted due to these issues, Weinberg and Bell's pioneering study established a new path in TEVG fabrication. In 2001, Shin'oka et al. conducted the first clinical trial of cell-seeded TEVGs. Autologous vascular cells were seeded on biodegradable grafts, which were then implanted in pediatric patients with congenital heart diseases [9]. To date, several clinical trials of biodegradable conduits, decellularized materials, and cell sheet-based TEVGs have been conducted [10–15]. These have shown promising results, yet TEVG application is still hindered by several limitations such as a lengthy fabrication period, high costs, thrombogenicity, and immunogenicity [16, 17].

Recent improvements in TEVG technologies have involved diverse cell sources (e.g., somatic and stem cells) and novel fabrication methods (e.g., scaffold-guided and self-assembled approaches). In this review, we provide an overview of recent advances in TEVG fabrication. We also propose a new manufacturing method in which supraphysiological periodic hydrostatic pressure promotes layered elastic fiber formation in TEVGs [18].

## 2. Mechanical Requirements of TEVGs

One of the important goals of TEVG development is to ensure sufficient mechanical strength for implantation. The required strength depends on the implantation site, but, in general, arterial shunts require more robust structure than venous shunts do. Although there is a lack of consensus regarding target strength, it has been common to use the saphenous vein burst pressure value (approximately 1680 ± 307 mmHg) for arterial shunts because the saphenous vein is a major autologous source for coronary bypass procedures [19]. An alternative target strength is the burst pressure value of the internal thoracic artery (3196 ± 1264 mmHg) [20] because, when implanted for coronary bypass, the thoracic artery has superior long-term patency compared to the saphenous vein [21]. Although it is more challenging to achieve the higher burst pressure of the internal thoracic artery, aiming toward this target strength may improve the long-term patency of TEVGs. Some advanced studies have already reported achieving burst pressures similar to that of the internal thoracic artery. L'Heureux et al. generated fibroblast-sheet-based TEVGs with a burst pressure of 3468 ± 500 mmHg [22], while Dahl et al. developed SMC-seeded biodegradable scaffold-based decellularized TEVGs with a burst pressure of 3337 ± 343 mmHg [23]. Details of their fabrication methods and implantation results will be described later.

In addition to graft strength, compliance is another important factor influencing the patency of TEVGs. Vascular compliance is influenced by functional and structural components [24]. Functional components involve neurohumoral elements, such as the renin-angiotensin system, the adrenergic nervous system, and EC-derived factors [24], which may become more important after graft implantation. Structural compliance is mainly determined by the presence and proportion of collagens and elastic fibers [24, 25]. While collagen determines the vascular stiffness at high pressure, elastic fibers are important in determining the stiffness at low pressure [24]. Compliance is expressed as a dimensional change with respect to luminal pressure change. The standardized testing method for compliance of tubular grafts was defined in ISO 7198: 2016 “Cardiovascular implants and extracorporeal systems—Vascular prostheses—Tubular vascular grafts and vascular patches” (https://www.iso.org/obp/ui/#iso:std:iso:7198:ed-2:v1:en). Commercially available vascular grafts have much lower compliance than native blood vessels do [26]; reported compliance values (%radial change per mmHg × 10^−2^) are as follows: femoral artery, 5.9 ± 0.5; saphenous vein, 4.4 ± 0.8; and ePTFE, 1.6 ± 0.2 [26]. Compliance mismatch between native vessels and vascular grafts is related to adverse biological responses including intimal hyperplasia [27, 28], which is the main cause of long-term graft stenosis. The change in resistance to pulsatile flow between compliant host artery and noncompliant graft leads to excessive mechanical stress resulting in wall injury [29]. Flow disturbance in anastomotic regions has a deleterious effect on ECs [27]. It is also reported that compliance mismatch causes greater intimal hyperplasia in end-to-side anastomosis than it does in end-to-end anastomosis because the end-to-side geometry is associated with increased intramural stress [28]. Reduction in compliance mismatch would improve the postoperative patency of TEVGs.

External mechanical forces also impact TEVG fabrication requirements and graft patency after implantation. These mechanical factors include cyclic stretch, hydrostatic pressure, and shear stress. Shear stress, for example, promotes nitric oxide production in ECs, which regulates vascular tone [30]. Pulsatile strain causes SMCs to secrete extracellular matrices including collagens [31] and tropoelastin [32]. Details of these cellular responses to external mechanical forces are described below.

## 3. Extracellular Matrix

Extracellular matrices (ECMs) are secreted from cells and form a three-dimensional network. Although the ECM is comprised of many proteins, its mechanical properties mainly depend on collagens and elastic fibers [25]. These ECMs support cells mechanically but also influence cell adhesion and thrombogenic properties [33, 34].

### 3.1. Collagen

The main structural proteins of the blood vessels are collagens, which are secreted from cells as precursor procollagens. Procollagens are converted into collagens by procollagen proteinases, and subsequently form collagen fibrils. Collagen fibrils are stabilized by lysyl oxidase-mediated crosslinking [35]. Ascorbic acid is a cofactor for the prolyl hydroxylase and lysyl oxidase, which promotes mature collagen fibers [36, 37]. Furthermore, ascorbic acid is reported to increase and stabilize collagen mRNAs [37]. Based on these findings, supplementation with ascorbic acid has been incorporated successfully into TEVG fabrication processes to increase collagen content and graft strength [19, 38].

In addition to their mechanical properties, collagens also have a cell recruitment feature via their integrin binding sites [33]. For example, collagen-based scaffolds have superior capacity for endothelialization compared to synthetic materials [39]. TEVGs with high-collagen content may have favorable effects on EC-recruitment.

When utilizing collagens in TEVGs, an important consideration is the attachment of platelets and coagulation proteins onto the integrin binding sites [40], which facilitates thrombus formation and coagulation processes. Huynh et al. constructed grafts using collagen biomaterials derived from the submucosa of the small intestine and type I bovine collagen [41]. This collagen-rich construct was crosslinked with 1-ethyl-3-(3-dimethylaminopropyl) carbodiimide hydrochloride, then treated with heparin-benzalkonium chloride complex to reduce thrombogenicity [41]. These treated collagen-based grafts were implanted into rabbit carotid arteries. Histological analysis revealed complete endothelialization 90 days after implantation without thrombosis [41]. These findings suggest that TEVGs with high-collagen matrices have greater strength and are able to recruit ECs, but their tendency to induce thrombosis must be controlled before they can be implanted [42].

### 3.2. Elastin

Elastic fibers consist of the core protein elastin and its surrounding microfibrils. Tropoelastin is secreted from cells as a precursor of elastin, and is crosslinked by lysyl oxidase to create insoluble elastic fibers [43]. In contrast to collagen fibers, elastic fibers are generated exclusively from the embryonic period through early childhood. Once elastic fibers are mechanically injured or degraded by inflammatory disease and/or aging, they rarely regenerate. Although limited studies have demonstrated increased levels of elastin in SMC-seeded TEVGs [44], it remains difficult to fabricate functional layered elastic fibers *in vitro*.

In addition to their mechanical properties, elastin and its degradation products have been reported to influence cell functions, including proliferation, adhesion, and chemotaxis [45]. The heterozygous loss-of-function mutation in the elastin gene *Eln* has been shown to cause excessive aortic SMC proliferation and subsequent aortic luminal obstruction in humans [46]. Similarly, elastin haploinsufficiency in mice causes increased SMC proliferation in the arteries, resulting in arterial wall thickening [47]. Thus, adequate elastic fiber formation is likely a key component in regulating vascular wall thickness through preventing aberrant SMC proliferation.

Elastic fiber formation, in contrast to collagen formation, reportedly exerts an antithrombogenic effect by decreasing platelet aggregation and thrombosis [34]. Simionescu et al. assessed the thrombogenicity of elastin using (i) a normal decellularized scaffold, (ii) a pure elastin scaffold, and (iii) a pure collagen scaffold [48]. Each of these scaffolds was obtained from porcine carotid arteries and subsequently decellularized with sodium dodecyl sulfate, cyanogen bromide, and a combination of dodecyl sulfate and elastase, respectively. Among these scaffold types, the elastin scaffold was associated with a decrease in platelet adhesion and aggregation *in vitro*. Furthermore, the implantation of the elastin scaffold into a rabbit carotid artery resulted in lower platelet adhesion compared to implantation of a polyvinyl chloride tube [48]. Hinds et al. evaluated the thrombogenicity of elastin-containing grafts comprised of an inner purified elastin layer and outer acellular small intestinal submucosa. The purified elastin layer was obtained from porcine carotid arteries that were treated first with 80% ethanol and then with NaOH solutions. This elastin layer was then wrapped with acellular small intestinal submucosa to increase graft strength. These elastin-containing grafts were implanted in porcine common carotid arteries, where they exhibited a longer patent period compared to commercially available ePTFE grafts [49]. These results suggest that incorporating elastic fibers into TEVGs might improve graft patency by reducing thrombogenesis.

## 4. Cell Sources

Within the blood vessel wall, the tunica media provides elasticity while the tunica intima plays an anti-inflammatory and antithrombogenic role. These layers are mainly composed of SMCs and ECs, respectively. The tunica adventitia, meanwhile, anchors the vessel to the surrounding tissue and is composed of various cell types including fibroblasts. It is important to consider each of these vascular cell types in the design and fabrication of functional TEVGs. Additionally, cellular origin is a crucial aspect that must be considered in TEVG implantation, given that allogenic cells cause graft rejection [50]. Although autologous cells are considered favorable graft sources, the isolation of autologous vascular cells requires invasive procedures and may be impossible to achieve in patients with systemic atherosclerosis. Furthermore, adult cells have limited proliferative capacity. To overcome these limitations, mesenchymal stem cells (MSCs) and induced pluripotent stem cells (iPSCs) are currently being investigated [51–53].

### 4.1. SMCs

SMCs can react to their surrounding environments and exert profound effects on ECM secretions [54]. This synthetic ability is particularly important in vascular development and remodeling as well as in the fabrication of TEVGs. Mechanical stresses induced by pulsatile blood flow, i.e., stretch and hydrostatic pressure, have been reported to influence SMC functions. Cyclic stretch increases the expression levels of SMC-specific differentiation markers [55], and SMC proliferation is dependent on stretch magnitude [55]. Mechanical stretch upregulates the expression levels of certain proteins in the ECM, including fibronectin [56], collagens [31], and tropoelastin [32]. In TEVGs, these SMC-stimulated ECM secretions are important to ensure sufficient graft strength. Their production can be triggered effectively through the use of bioreactors in TEVG fabrication [38].

In contrast to the effects of stretch, the effects of hydrostatic pressure on vascular cells are less well understood, although hydrostatic pressure does appear to influence SMC characteristics. Some reports have demonstrated that increased hydrostatic pressure promotes SMC proliferation [57, 58]. We have demonstrated that periodic hydrostatic pressurization promotes actin stress fiber formation and fibronectin fibrillogenesis in vascular SMCs, a process that has allowed us to generate implantable TEVGs as described below [18]. Thus, both cyclic stretch and hydrostatic pressure affect SMC differentiation and proliferation and ECM synthesis in ways that are highly relevant to TEVG production.

### 4.2. ECs

ECs play major roles in antithrombogenicity and in combating bacterial and viral invasion [59]. TEVGs containing layered ECs are resistant to thrombosis and bacterial attachment, which improves their graft patency [42].

It is also well recognized that ECs sense blood flow-induced shear stress. This EC-induced mechanoresponse contributes to several vascular functions. ECs have been found to regulate vascular tone and blood coagulation by producing nitric oxide in response to shear stress [30]. ECs also regulate inflammatory responses by inducing increased expression and/or activation of proinflammatory signaling in response to chronically disturbed blood flow [60], while inducing an anti-inflammatory response when shear stress is not disturbed [61].

A previous report investigated the impacts of different flow patterns on ECs. Gong et al. seeded rat aortic ECs on sulfated silk fibroin nanofibrous scaffolds and compared the effects of three different flow patterns: steady laminar flow, sinusoidal flow, and physiological pulsatile flow [62]. Among these patterns, physiological pulsatile flow was associated with higher EC retention, much greater F-actin rearrangement and fibronectin fiber formation, and a lower apoptosis ratio compared to the other flow patterns [62]. These findings suggest the importance of physiological pulsatile shear stress in the fabrication of EC-seeded TEVGs.

Seeding vascular-derived ECs may enable the production of functional TEVGs. It can be difficult, however, to obtain sufficient autologous ECs due to their limited proliferative capacity. In addition, it has been reported that more than 90% of seeded ECs on grafts are lost due to hemodynamic stress after implantation *in vivo* [63]. Although some studies have reported that culturing ECs under shear stress prior to EC seeding into a scaffold improved the settling of ECs on grafts [64], promoting *in vivo* endothelialization using host-derived ECs may also be useful [42].

To overcome the limited availability of autologous vascular-derived ECs, endothelial outgrowth cells (EOCs, also known as endothelial colony forming cells) have recently been extensively investigated [65]. Endothelial progenitor cells in the peripheral blood give rise to EOCs, which subsequently become mature ECs [66]. EOCs can be isolated from blood and show a high proliferation rate compared to vascular-derived ECs [67]. Glynn and Hinds obtained pairs of donor-matched carotid ECs and EOCs from baboons and compared their antithrombogenic and anticoagulant properties [68]. *In vivo* experiments showed that the expression levels of genes involved in thrombogenic and inflammatory responses were highly similar between ECs and EOCs at basal conditions and following inflammatory stimulus [68]. Furthermore, EC- and EOC-seeded ePTFE grafts demonstrated the same levels of platelet accumulation and fibrinogen incorporation in an *ex vivo* baboon femoral arteriovenous shunt loop [68]. EOCs, which can be noninvasively isolated from the peripheral blood, may be well suited for a vascular tissue engineering application.

### 4.3. Fibroblasts

Fibroblasts are a major cell type in the tunica adventitia [69]. Like SMCs, fibroblasts can react to their surrounding environments and secrete ECM proteins including collagens of various types [70]. The organized structure and high-collagen content of the adventitia prevents vascular rupture at high blood pressures [25]. Fibroblasts have the ability to differentiate into myofibroblasts, which produce more collagens than quiescent fibroblasts upon stimulation with transforming growth factor-*β*1 (TGF*β*1) in conjunction with high tensile stress [71, 72]. In contrast to the rapid contraction and relaxation seen in SMCs, myofibroblasts exhibit a long-lasting isometric tension resulting in a slow and irreversible retraction [73]. Fibroblast-derived ECMs have been successfully used to support the strength of TEVGs [22, 74].

### 4.4. MSCs

Mesenchymal stem cells (MSCs) are one of the more promising possible cell sources for TEVGs because of their high regenerative ability, immunoregulatory and antithrombogenic features, and transdifferentiation capacities. MSCs have been isolated from bone marrow, adipose tissues, peripheral blood, and even blood vessel walls [75]. The number of population doublings is much higher among MSCs than among adult somatic cells [76]. MSCs have been reported to interact with allogenic immune cells, including dendritic cells, T cells, and natural killer cells, causing these cells to decrease production of proinflammatory cytokines and increase production of suppressive cytokines, and thereby suppressing allogenic immune responses [77].

MSCs have antithrombogenic properties because the heparan sulfate proteoglycans expressed on their cell surfaces attenuate platelet adhesion [78]. It has also been reported that MSCs recruit ECs through the secretion of angiogenic factors [79]. Zhao et al. have fabricated cell sheet-based TEVGs from MSCs [51]. These MSC grafts were implanted in rat abdominal aortas and showed complete endothelialization four weeks after implantation.

MSCs can also be transdifferentiated into both SMCs [80] and ECs [81]. Gong and Niklason have integrated the differentiation of MSCs into SMCs into their biodegradable polymer-based TEVG production process [82]. Undifferentiated MSCs were seeded on fibronectin-coated biodegradable PGA scaffolds and cultured under static conditions for four weeks. The MSC-seeded TEVGs were then supplemented with TGF*β*1, which is reported to induce the differentiation of MSCs into SMCs [80], and exposed to pulsatile flow. After four weeks of pulsatile incubation, the MSCs in these fabricated TEVGs had successfully differentiated into SMCs, as confirmed by the expressions of smooth muscle *α*-actin (SMA) and calponin as well as by their morphological characteristics [82].

### 4.5. iPSCs

Recently, iPSCs have attracted considerable attention with regard to their usefulness in the generation of neotissues including vascular grafts. Because they are derived from reprogrammed somatic cells, iPSCs are not subject to ethical constraints, unlike embryonic stem cells. iPSCs can be generated from a patient's own cells, eliminating the problem of immune rejection due to autologous transplantation. To utilize iPSCs *in vivo*, it is necessary to induce iPSCs to differentiate into specific lineages because their pluripotent properties otherwise make them capable of producing a teratoma. Like MSCs, iPSCs can also be differentiated into SMCs and ECs [83, 84]. Luo et al. fabricated TEVGs containing human iPSC-derived vascular SMCs [53]. Human iPSCs were differentiated into vascular SMCs using an embryoid body-based approach, in which it takes 26 days to obtain mature vascular SMCs [53]. Differentiated SMCs were seeded onto a biodegradable scaffold and incubated under pulsatile flow provided by a bioreactor for eight weeks. The resulting fabricated TEVGs showed layered SMC populations, as confirmed by the expressions of SMA, calponin, and smooth muscle myosin heavy chain [53].

It is important to note that iPSCs are likely to spontaneously differentiate into cell lineages of all three germ layers, leading to heterogenous cell populations. Even if specific induction protocols are applied, induction efficiency remains an issue. Accordingly, further studies are aimed at optimizing induction protocols to enable us to obtain much needed pure cell populations [85].

## 5. Fabrication Methods

**Figure 1:**
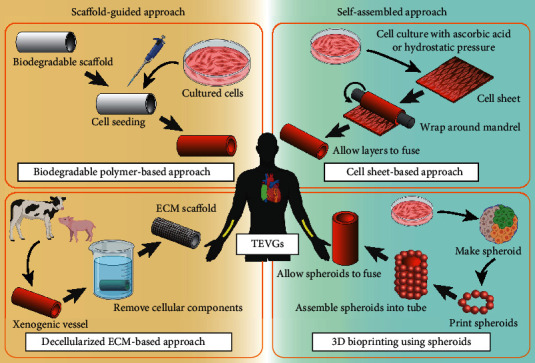
Schematic representation of TEVG manufacturing methods. Four representative fabrication methods are depicted. TEVGs fabricated as depicted in the right (green) panels are self-assembled approaches, while TEVGs fabricated as depicted in the left (yellow) panels are scaffold-guided approaches. TEVGs fabricated according to these methods are intended as either large-diameter venous shunts in pediatric patients with congenital heart disease (placement shown in green in human schematic) or small-diameter arteriovenous shunts in adult patients with renal disease (placement shown in yellow). This review introduces the spheroid-based technique as a novel application of 3D bioprinting (lower right panel). Graphical objects were created with BioRender (https://biorender.com/).

To date, several fabrication methods for TEVGs have been developed, including (i) a biodegradable polymer-based approach, (ii) a decellularized ECM-based approach, (iii) a cell sheet-based approach, and (iv) three-dimensional (3D) bioprinting (Figure 1). The first two approaches are classified as scaffold-guided approaches, while the cell sheet-based approach is classified as a self-assembled approach without synthesized materials. 3D bioprinting, which uses relatively recent technology, has diverse potential applications. Among the many possibilities, this review focuses on a spheroid-based technique, which is one of the self-assembled approaches. Current progress with other 3D printing techniques in the field of cardiovascular tissue engineering have been described in other excellent reviews [86, 87].

TEVGs can be divided into two categories according to their target diameters: small (e.g., arteriovenous shunt and coronary arteries) and large (e.g., great arteries and veins). A biodegradable polymer-based approach has been applied to both small- and large-diameter TEVGs, while the other approaches have so far focused on producing small-diameter grafts.

The time it takes to fabricate implantable TEVGs varies among methods, but it generally takes weeks or months. TEVGs are therefore considered more suitable for use in planned or elective surgeries rather than emergency surgeries. It is worth noting that decellularized materials may be the most readily available for use in emergency situations because some decellularized materials can be cryopreserved until an operation.

### 5.1. Biodegradable Polymer-Based Approach

TEVGs need to be able to withstand the blood pressures to which they will be exposed at their implanted sites. The cells in implanted TEVGs eventually begin to secrete and organize their own ECMs, which provide them with sufficient mechanical properties. Yet because this process requires considerable time, it is necessary to support the implanted cells until the ECM networks are organized. Biodegradable polymers are designed to function as temporary scaffolds that provide mechanical support to seeded vascular cells. Polyglycolic acid (PGA), polylactic acid (PLA), poly(*ε*-caprolactone) (PCL), and their combinations are most frequently used as biodegradable materials for TEVGs [88, 89]. Their degradations are caused by cleavage of the ester bond. The *in vivo* durations of PGA, PLA, and PCL prior to complete degradation are two to three weeks, six to 12 months, and two to three years or more, respectively [88, 89]. Their degradation rate depends on the size, surface area, and composition of the materials. By changing these factors, their mechanical properties and degradation rate can be manipulated. Biodegradable scaffolds with longer degradation times are able to retain their mechanical strength and withstand blood pressure for longer periods, although this may hinder host-derived neotissue formation after implantation. An imbalance between material degradation and neotissue formation leads to mechanical mismatch and subsequent graft failure [90]. In addition, the acidic products from the breakdown of PGA scaffolds have been reported to induce SMC phenotypic changes, resulting in a synthetic phenotype, which leads to SMC proliferation and subsequent luminal narrowing [91].

SMC-seeded TEVGs were initially reported by Yue et al. in 1988 [92]. Rat vascular SMCs were seeded on biodegradable polyurethane-based scaffolds and implanted in the rat aorta. The implanted grafts were harvested at two hours, two days, and one week after implantation. Multilayered SMCs were first observed at two days after implantation and had become thicker by one week after implantation. The neotissues showed EC-like cells on the luminal side. These histological changes were not observed in control grafts without SMC seeding.

In 1997, Niklason et al. generated SMC- and EC-seeded TEVGs using bioreactor systems [38, 93]. Their bioreactor was composed of a pulsatile pump, a buffer reservoir, and a glass bottle with an inlet/outlet of compliant silicon tubing [94]. To fabricate the TEVGs, a biodegradable PGA tube was first connected to the inlet/outlet of the silicon tubing in the glass bottle. Then, bovine aortic SMCs were seeded onto the PGA tube, which was carefully rotated to allow cells to attach evenly over the surface. After 30 min of incubation, the glass bottle was filled with culture medium. An external pulsatile pump was connected to the inlet of the silicon tubing, through which pulsatile flow was transmitted into the lumen of the PGA tube. The reservoir provided the basal pressure to the lumen of the PGA tube. The pulsatile radial strain was 165 beats per minute. After eight weeks of pulsatile incubation, ECs were applied to the lumen, and the graft was then exposed to continuous perfusion for three days.

Niklason et al. assessed the effect of their bioreactor by comparing pulsed-incubated TEVGs to static-cultured TEVGs in 1999 [38]. While static-cultured TEVGs spontaneously ruptured at 270 mmHg after seven weeks of incubation, the burst pressure of pulsed TEVGs gradually increased, reaching 570 ± 100 mmHg after five weeks of incubation and 2150 ± 709 mmHg after eight weeks. The authors also confirmed by histological analysis that SMCs were distributed as a layered structure with interposed layered collagen fibrils [95]. The collagen weight content after eight weeks of incubation was similar to that of native vessels, while those of grafts not exposed to pulsatile incubation were low. Furthermore, Niklason's research group recently improved their bioreactor from one that provides uniaxial loading to one that provides biaxial loading [44]. In addition to the previous uniaxial strain caused by the pulsatile pump, the new bioreactor is furnished with a linear motor contractor, which enables the addition of a periodic stretching of the PGA tube by up to 8% of its length at 0.0333 Hz. Exposure to biaxial stretching resulted in the formation of some elastic fibers and undulated collagen fibers, resulting in a high suture retention strength (static: 128 ± 14 g; uniaxial: 243 ± 13 g; and biaxial: 303 ± 15 g) [44].

In 2014, Sundaram et al. and Luo et al. reported the fabrication of TEVGs from human iPSC-derived SMCs [52, 53]. They seeded iPSC-derived SMCs on biodegradable PGA scaffolds; then, they incubated them under pulsatile radial strain induced by a bioreactor. The mechanical properties of TEVGs generated from iPSC-derived SMCs were relatively weak (burst pressure: 500-700 mmHg) [52]. In a subsequent study, however, the groups further optimized the bioreactor settings and the proportions of growth factors added during culture of iPSC-derived SMCs. Under the optimized conditions, the pulse rate was lowered from 165 to 115 beats per minute, and radial distention was increased from 1-2.5% to 3%. In addition, the cell growth factor recipe was modified as platelet-derived growth factor-BB was removed. These modifications increased the mechanical strength of TEVGs (1419 ± 174 mmHg), which were successfully implanted in nude rat aortas without apparent disadvantages [53].

Biodegradable polymer-based TEVGs have also been investigated by other researchers. In 2009, Vorp's research group developed a biodegradable bilayered elastomeric scaffold [96]. This bilayered scaffold consists of a highly porous inner layer, which allows cell integration and growth, and an external reinforcing fibrous layer [96]. Human skeletal muscle-derived stem cells (MDSCs) were seeded on the inner surface of the scaffold using a rotational vacuum seeding system [97]. In this seeding system, a cell suspension was infused into the manually rotating tubular scaffold, which was also connected to an external vacuum to maintain a negative pressure, inducing transmural flow across the scaffold. Two minutes after cell seeding, the scaffold was placed in a static culture for three hours to allow cell attachment, and was then transferred to a spinner flask with culture medium which was stirred at 15 revolutions per minute (rpm) for seven days [96]. The resulting TEVGs showed high cell density throughout the inner layer and high burst pressure comparable to that of the human saphenous vein [96]. Later, Vorp's research group seeded human pericytes [98] and rat MDSCs [99] into their bilayered biodegradable scaffolds. These cell-seeded TEVGs were successfully implanted into rat abdominal aortas, where they showed 100% and 65% patency, respectively, at eight weeks after implantation [98, 99].

A 2007 study utilized the antithrombotic property of undifferentiated MSCs on biodegradable poly(L-lactic acid) (PLLA) materials [78]. TEVGs generated from undifferentiated MSCs showed reduced intimal hyperplasia and persistent patency for eight weeks after implantation in the rat common carotid artery. In 2008, Mirza et al. seeded undifferentiated MSCs on polyurethane vascular prostheses, then implanted them in rat aortas [100], and found that the MSCs, which they had labeled with green fluorescence protein (GFP), differentiated into SMC-like cells *in vivo*. These data suggest not only a promising future for MSC-seeded scaffold-based TEVGs, but also the importance of mechanical and biological environments in ensuring appropriate cell differentiation.

Hoerstrup's research group implanted autologous myofibroblast- and EC-seeded PGA scaffolds (internal diameter 18 mm) into growing lambs in 2006 and 2010 [101, 102]; the results suggested that cell-seeded biodegradable polymer-based TEVGs have potential for growth. Autologous myofibroblasts were seeded on PGA three times at 24-hour intervals, followed by EC seeding. Three days after static culture, this construct was transferred to a pulsatile bioreactor and incubated for 21 days. Fabricated TEVGs were implanted as main pulmonary artery replacements, and followed up for two years after implantation. Implanted lambs grew to more than double their original body weight over the two years of follow-up. Implanted grafts showed a 30% increased diameter and a 45% increased length compared to their original dimensions. Computed tomography-angiography revealed no evidence of stenosis, calcification, or aneurysm, and echocardiography showed no stenotic flow [101].

### 5.2. Decellularized ECM-Based Approach

Decellularized matrices are obtained from the 3D structures of tissue ECMs. To avoid adverse immunological responses, cellular components must be completely removed from the tissues [103]. Detergents are widely used for decellularization purposes because they solubilize the lipid bilayer of cell membranes and dissociate DNA from protein, effectively removing cellular components [104]. Hypotonic and hypertonic solutions can also be utilized. Hypotonic solutions cause cell lysis through simple osmotic effects, while hypertonic saline dissociates DNA from proteins [104]. The freezing and thawing procedure also can be utilized to lyse cells. While freezing and thawing methods are effective for preserving ECM components (e.g., collagen, elastin, and fibronectin) and mechanical strength compared to detergent methods [105], it must be followed by other decellularization methods because freezing and thawing alone cannot remove all cellular components (e.g., 90% of DNA) [106]. Trypsin has frequently been used for enzymatic decellularization [106], but it was reported to have disruptive effects on ECMs compared to detergent treatments [107]. The most effective method varies based on the original tissue's thickness, cellularity, density, and lipid contents. Of note, all decellularization methods cause some degree of ECM alteration and structural disruption [107], and the resulting protein alterations may affect the mechanical and biological properties of ECMs.

Dahl et al. assessed the effects of three decellularization methods using porcine carotid arteries in 2003 [108]. The methods utilized were as follows: in Treatment A, vessels were submerged in a nonionic detergent solution (Triton X-100) with a chelator (ethylenediaminetetraacetic acid) to inhibit enzymes and deoxynuclease for 24 hours; in Treatment B, vessels were submerged in a hypotonic solution for 11 hours and then transferred to a hypertonic solution for 11 hours; and in Treatment C, vessels were submerged in a zwitterionic detergent solution (3-[(3-cholamidopropyl)dimethylammonio]-1-propanesulfonate) for 11 hours and then transferred to an anionic detergent solution (sodium dodecyl sulfate) for 11 hours. Among these methods, Treatment C resulted in the most efficient removal of the nuclei in vessels. Although all decellularized vessels showed slightly lower burst pressure and greater compliance compared to native vessels, Treatment C resulted in mechanical properties that most closely resembled those of the native vessels. Dahl et al. accordingly used Treatment C in their subsequent studies.

In 2011, Dahl et al. reported that they had generated biodegradable scaffold-based decellularized TEVGs [23]. Allogenic aortic SMCs were seeded on biodegradable PGA scaffolds. After seven to 10 weeks of incubation under cyclic strain by a bioreactor, fabricated TEVGs were decellularized according to Treatment C. The decellularized TEVGs showed a burst pressure of 3337 ± 343 mmHg, which could be preserved at a temperature below 4°C for up to 12 months. These decellularized TEVGs were seeded with autologous ECs shortly before implantation. The TEVGs were then implanted as arteriovenous conduits in a baboon model, where they maintained 88% patency without aneurysmal formation for up to six months.

### 5.3. Cell Sheet-Based Approach

Although scaffold-guided approaches have improved over time, the issues regarding the biocompatibility of exogenous materials remain. Ideally, TEVGs can be remodeled by living cells based on their surrounding environments. In scaffold-based TEVGs, this remodeling process may be disrupted by the breakdown products of biodegradable materials [91] and the denatured proteins of decellularized ECMs [107].

Self-assembled approaches without scaffolds have been considered to offer theoretical advantages, such as allowing cells to form cell-cell junctions and build natural ECM networks. L′Heureux et al. generated the first cell sheet-based TEVGs without any synthetic or exogenous biomaterials in 1998 [19]. They cultured human umbilical vein SMCs and human skin fibroblasts with ascorbic acid, which enhances collagen synthesis, for four weeks, and observed abundant ECM production in the resulting TEVGs. The SMC sheets were manually peeled and wrapped around the mandrel to mimic the tunica media. These SMC media-mimetic constructs were cultured in a bioreactor with pulsatile flow for one week. Fibroblast sheets were similarly manufactured and wrapped around the SMC media-mimetic construct to mimic the tunica adventitia. These constructs were cultured for an additional seven weeks in a bioreactor. Human umbilical vein ECs were then seeded to the inner side of each construct. Each layer was fused together one week after the ECs were seeded. A total of four months were required between the start of the procedure and implantation. The burst pressure was 2594 ± 501 mmHg, which is comparable to that of the human saphenous vein. These grafts were implanted in the canine femoral artery for a short-term evaluation. In this animal study, EC seeding was omitted to avoid acute rejection due to the problems associated with xenografts. Although the graft patency was 50% at seven days after implantation due to thrombus formation, the grafts were shown to be well sutured and to withstand blood pressure *in vivo* [19].

Furthermore, L'Heureux et al. advanced their cell sheet-based technique to incorporate patient-derived fibroblasts that were obtained by skin biopsy from coronary bypass patients [22]. The resulting advanced TEVGs were composed of three layers: a living-fibroblast-derived adventitia, an acellularized middle layer, and an inner EC layer. First, the fibroblast sheet was constructed during eight weeks of incubation. This fibroblast sheet was wrapped around the mandrel and then incubated for 10 weeks as it fused into homogenous cylindrical tissue. This tissue was then dehydrated to form an acellular construct. This acellular construct was wrapped with a new fibroblast sheet in the same fashion. After this second maturation, the mandrel was removed and ECs were seeded in the lumen. These three-layered TEVGs were then placed in a pulsatile bioreactor. Fabrication time ranged between six and nine months until implantation. The burst pressure in the resulting grafts was 3468 ± 500 mmHg. These TEVGs were implanted in rats and primates for long-term evaluation. In rats, the TEVGs were implanted in the nude rat aorta and evaluated for up to 225 days after implantation. Histological analysis revealed 86% patency and neomedia formation. In primates, the TEVGs were implanted in the iliac artery or abdominal aorta of immunosuppressed cynomolgus primates as interpositional grafts; they maintained 100% patency without aneurysm formation for up to eight weeks after implantation.

Bourget et al. fabricated SMC sheet-based TEVGs in 2012 [74]. They used decellularized fibroblast-derived ECMs as the scaffolds for SMC-seeded TEVGs. In this study, a human donor-derived saphenous vein or dermal fibroblasts were cultured with ascorbic acid for three weeks. This fibroblast sheet was decellularized by osmotic shock, in which the fabricated sheet was rinsed three times with deionized water for 20 min, kept overnight in water at 4°C, and then dried at room temperature for eight hours. Human umbilical artery SMCs were seeded on this fibroblast-derived decellularized matrix. After one week of incubation, SMC sheets were manually detached from the culture flank and wrapped around the mandrel. After three additional weeks of incubation, this method yielded TEVGs generated from SMC sheets with fibroblast-ECM. This method shortened the fabrication time to four weeks from the six weeks required to produce TEVGs from SMC sheets without fibroblast-ECM. Moreover, these SMC sheet-based TEVGs with fibroblast-ECM showed greater resistance to tensile load than SMC sheet-based TEVGs without fibroblast-ECM did.

### 5.4. 3D Bioprinting

3D printing was initially developed as a means of fabricating 3D molds and scaffolds with arbitrary material properties [86]. Since then, 3D printing has advanced to allow “3D bioprinting,” which utilizes cells as a type of bioink [87]. The most common bioink is cell-laden hydrogels, in which hydrogels act as a scaffold for supporting cells. More recently, spheroids, collections of thousands of single cells, have been developed as a bioink for use in 3D bioprinting. Spheroids are considered an ideal bioink because a spheroid allows cells to form cell-cell junctions and assemble their own ECM [87]. Although clinically available bioprinted TEVGs are not yet available, 3D bioprinting technology is improving rapidly.

Norette et al. performed an initial study attempting to bioprint small-diameter vasculature from spheroids in 2009 [109]. They utilized spheroids of human skin fibroblasts that were bioprinted on agarose-based rods. These agarose rods were removed from the printed grafts after seven days of incubation. Norette et al. also demonstrated the construction of a double-layered vascular tube composed of an inner SMC layer generated from spheroids of human umbilical vein SMCs and an outer fibroblast layer generated from human skin fibroblasts [109].

Nakayama's research group generated implantable scaffold-free bioprinted TEVGs in 2015 [110]. They generated multicellular spheroids composed of human umbilical vein ECs (40%), human aortic SMCs (10%), and human dermal fibroblasts (50%). They used a needle array rather than a hydrogel mold. Multicellular spheroids were skewered into the needle array where they formed the tubular constructs. After four days of incubation, the needle array was removed, and the tubular construct was perfused with a bioreactor for an additional four days. The resulting TEVGs were successfully implanted in the abdominal aortas of nude rats, where they maintained patency until the end point of the study on postoperative day 5. Histological analysis revealed endothelialization after implantation. More recently, Nakayama's research group has reported a method of cryopreserving spheroids using 10% Me_2_SO_4_ solution [111]. Cryopreserved spheroids were printed on a needle array. Although cryopreserved spheroids had 17% less cell viability compared to non-cryopreserved spheroids and the resulting tubular constructs exhibited lower tensile strength, the cryopreservation methods demonstrated in that study may be useful in reducing the wait time involved in cell expansion and large-scale spheroid creation, indicating the potential feasibility of 3D bioprinted TEVGs for clinical applications [111].

## 6. Clinical Trials of Each Method

Clinical trials have been conducted on TEVGs produced according to each of the fabrication methods described above. Trials have investigated large-diameter TEVGs made using a biodegradable polymer-based approach for venous shunts in pediatric patients with congenital heart disease [9, 10] and small-diameter TEVGs for arteriovenous shunts in adult patients undergoing hemodialysis [11–15, 112, 113]. These trials have achieved promising results, although intimal hyperplasia and thrombosis are still major complications of TEVG implantation [9–15, 112, 113].

### 6.1. Biodegradable Polymer-Based Approach for Venous Shunt

The first successful clinical application of biodegradable scaffolds was conducted by Shin'oka et al. in 2001 [9]. Shin'oka et al. explanted autologous vascular cells from an autologous peripheral vein, and seeded these cells on a biodegradable scaffold composed of a 50 : 50 copolymer of PLA and PCL. The resulting TEVG with autologous vascular cells was implanted as an extracardiac cavopulmonary conduit for corrective surgery for congenital heart disease. The implanted site was a large-diameter venous system with low pressure and high blood flow. Although their initial operation was successful, the required time for cell expansion was eight weeks. The same group used bone marrow cells in TEVGs for their next clinical trial, in which 25 patients underwent extracardiac total cavopulmonary connection surgery between 2001 and 2004 [10]. During follow-up (average duration: 11 years), there was no graft rupture, infection, aneurysm, or calcification. The most frequent problem was graft stenosis, as 28% of the patients required balloon angioplasty for asymptomatic graft stenosis [114].

### 6.2. Decellularized ECM-Based Approach for Arteriovenous Shunt

At this writing, several decellularized grafts originating from bovine carotid artery (Artegraft®), bovine mesenteric vein (Procol®), bovine ureter (SynerGraft®), and human femoral vein (Cryovein®) are commercially available. Katzman et al. conducted a multicenter prospective trial of Procol® for hemodialysis access between 1999 and 2002 [12]. The study included both decellularized grafts (*n* = 183) and classical ePTFE grafts (*n* = 93). Decellularized grafts exhibited primary patency rates equivalent to those of ePTFE grafts (35% vs. 28% at 12 months) with lower rates of complications, including dilatation, infection, and thrombosis, compared to conventional ePTFE grafts.

Chemla and Morsy conducted a randomized clinical trial of SynerGraft® for hemodialysis grafts between 2001 and 2005 [13]. Their study revealed that decellularized grafts (*n* = 29) had comparable graft patency compared to ePTFE (*n* = 27) (28% vs. 48% at 12 months) and a similar ratio of freedom from infection (96% vs. 91% at 12 months). These clinical trials suggest that decellularized grafts generally have a patency rate similar to those of conventional prosthetic grafts.

### 6.3. Biodegradable Polymer and Decellularized ECM: Combined Approach for Arteriovenous Shunt

Niklason's research group conducted a phase 2 clinical trial of small-diameter biodegradable PGA scaffold-seeded allogenic SMCs between 2012 and 2014 [11]. Biodegradable PGA scaffolds were seeded with organ donor-derived SMCs and incubated under pulsatile strain applied by a bioreactor [38]. After eight weeks of pulsatile incubation, the resulting TEVGs were decellularized to remove allogenic antigens using a zwitterionic detergent and an anionic detergent [23]. Seven days before implantation, the lumens of these decellularized TEVGs were seeded with autologous ECs. These TEVGs were then implanted as arteriovenous dialysis shunts in a cohort of 60 patients with end-stage renal disease. Their successful implantation and one-year follow-up revealed no immune rejection, aneurysmal formation, or postcannulation bleeding. The primary patency was 28% at 12 months after implantation [11], which was similar to that reported for ePTFE [115]. Small graft segments were obtained from eight patients at 16-55 weeks after implantation. Histological analysis of the obtained postoperative samples showed luminal endothelialization and repopulation of vascular SMCs in the implanted graft walls. Currently, two additional phase 3 clinical trials comparing these TEVGs to ePTFE and arteriovenous fistulas are ongoing [116].

### 6.4. Cell Sheet-Based Approach for Arteriovenous Shunt

The first clinical trial of cell sheet-based TEVGs as hemodialysis grafts was conducted by L'Heureux's research group between 2004 and 2007 [14, 15]. Their clinical trial involved 10 patients with end-stage renal disease. Autologous fibroblasts and ECs were extracted by biopsy from the skin (about 2 cm^2^) and superficial veins (about 3 cm long) of each patient. The TEVGs generated for this trial contained three layers, namely, a fibroblast-derived adventitia, an acellularized middle layer, and an inner EC layer, as described above [22]. These three-layered TEVGs were subjected to pulsatile strain applied by a bioreactor; the total generation time for this method ranged from six to nine months. The burst pressure of the resulting TEVGs was 3512 ± 873 mmHg. After they were implanted as arteriovenous shunts, three of the 10 patients experienced early graft failure. Another patient was withdrawn due to gastrointestinal bleeding, and another died of unrelated causes. The grafts in the five remaining patients functioned for 6-20 months. Their primary patency was 78% at one month after implantation and 60% at six months after implantation.

For their next clinical trial, L'Heureux's research group generated cell sheet-based TEVGs from allogenic fibroblasts [112, 113]. Since fibroblasts in culture do not express major histocompatibility complex class II antigens [117], decellularization prior to implantation is believed to be less important when fibroblasts are used. Accordingly, the researchers chose to use devitalization, a less thorough decellularization method. After eight weeks of incubation with ascorbic acid, allogenic fibroblast sheets were constructed and wrapped around mandrels. During an additional 10 weeks of incubation, the cell sheets developed into cylindrical tissues. They were subsequently dehydrated and stored at -80°C for six to nine months. These constructs were rehydrated a few days prior to implantation. L'Heureux's research group reported two case studies involving these fibroblast sheet-based TEVGs; the first study seeded autologous ECs on the lumens of the grafts (*n* = 1) [113], while the second study did not use ECs (*n* = 3) [112]. All grafts were successfully implanted as arteriovenous shunts, and there was no evidence of adverse immune responses. Although thrombogenic failures occurred three to five months after implantation in two out of the three grafts implanted in the second case study, there was no evidence of early thrombosis formation in an earlier postimplantation stage. Further study is required to delineate the utility of allogenic fibroblasts and the necessity of EC seeding for preventing thrombogenic failure.

## 7. A Novel Approach to Fabricating TEVGs with Layered Elastic Structure

As described above, several TEVG fabrication methodologies have been developed, and several clinical trials have shown their potential for clinical use [10–14]. However, the advantages of TEVGs compared to conventional vascular grafts have not yet been established [16, 17]. A major issue related to their long-term clinical outcomes is the possibility of graft stenosis, which presently affects scaffold-guided TEVGs [11, 13, 114] as well as conventional ePTFE grafts. The main cause of graft stenosis is intimal hyperplasia, which is associated with compliance mismatch between implanted grafts and native vessels [27, 28]. Graft compliance is determined by ECMs, i.e., collagen and elastic fibers. While collagen synthesis in TEVGs has been improved by incorporating bioreactor incubation and ascorbate supplementation into fabrication processes [19, 38], elastic fiber synthesis in TEVGs is still insufficient. Thus, the fabrication of vascular grafts containing organized elastic fiber structures remains a challenging issue in TEVG production.

Our group demonstrated TEVGs containing layered elastic fiber formation by fibronectin coating to SMCs [118, 119]. A fibronectin meshwork on the cell surface is essential for subsequent microfibril formation, which is required for elastic fiber formation in the proper arrangement [43]. We immersed monolayered rat neonatal aortic SMCs alternatively into fibronectin and gelatin solution, then seeded the next SMC layer onto the first layer of SMCs [118]. We repeated this procedure every 12 hours, up to seven times in total. This procedure resulted in the successful regeneration of layered elastic fiber formation in TEVGs *in vitro* [118].

Mechanical stimulation has been suggested to play crucial roles in vascular tissue development [25] and in the maintenance of vascular cell functions throughout life [70]. In the field of TEVG development, the use of bioreactors for tissue incubation has become an important step in obtaining sufficient graft strength by increasing ECM production [38]. In addition to stretch and sheer stress, hydrostatic pressure is also a major mechanical stress on vasculature. Yet only a few reports have discussed the application of hydrostatic pressure in TEVG fabrication. In the tissue culture of a porcine aortic valve, collagen synthesis was increased by hydrostatic pressure in a magnitude- and frequency-dependent manner [120]. Hydrostatic pressure also increased elastin secretion and deposition in biodegradable poly(glycerol sebacate) (PGS) scaffolds seeded with adult baboon arterial SMCs [121]. Although this previously reported evidence suggests that hydrostatic pressure is related to ECM network formation, a reliable means of stimulating hierarchically organized elastic fiber formation has not been established. We hypothesized that periodic hydrostatic pressurization simulating blood flow would stimulate layered elastic fiber formation. To confirm this, we investigated the effect of hydrostatic pressure on TEVG fabrication.

**Figure 2:**
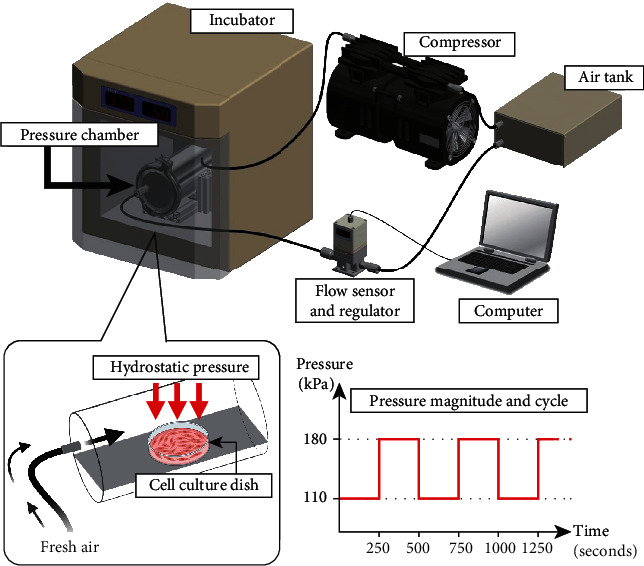
A periodic hydrostatic pressurization system. Our experimental system was composed of a pressure chamber containing a cell culture dish, a compressor for collecting 37°C air containing 5% CO_2_ from the incubator and transferring it to an air tank, a flow sensor and regulator for controlling the flow of pressurized air into the pressure chamber, and a computer for setting the desired magnitude and frequency of the periodic pressurization. Pressurization to 110-180 kPa at a frequency of 0.002 Hz is illustrated.

We developed a culturing system that enables us to culture cells under periodic hydrostatic pressurization (PHP) for long periods (Figure 2) [18]. This system is composed of a pressure chamber, an incubator, a compressor, a flow regulator, a pressure sensor, an air tank, and an external computer. The compressor inlet is connected to the incubator containing air at 37°C with 5% CO_2_, and the outlet is connected to the air tank. Once air in the incubator is collected in the air tank, pressurized air is supplied to the pressure chamber by regulating the flow from the tank. The magnitude and frequency of the pressure are controlled by the computer. The tunable ranges of frequency and magnitude are 0-0.25 Hz and 101-1000 kPa, respectively. We applied biphasic pressure to the cultured cells, which enabled us to circulate fresh air containing 5% CO_2_ into the pressure chamber during pressurization [18]. The pressure chamber was placed inside the incubator and kept at 37°C during the experiments.

We examined the effect of PHP at various magnitudes and frequencies on human umbilical artery SMCs [18], which can be obtained in a noninvasive manner. In humans, systolic blood pressure is approximately 110 mmHg and heart rate is 1.2 Hz (70 beats per minute). However, we unexpectedly found that supraphysiological PHP settings, namely, a pressure of 110 to 180 kPa (65 to 590 mmHg) with a cycle of 0.002 Hz, significantly increased fibronectin expression and fibronectin fibrillogenesis in vascular SMCs within 24 hours [18]. Furthermore, actin cytoskeleton polymerization and elastic fiber-related genes were also increased under these conditions [18]. The PHP-induced fibronectin fibrillogenesis and actin stress fiber formation were attenuated by inhibition of Rho/Rho-kinase [18], which has been reported to be involved in the mechanotransduction of MSCs [122]. We also found that adding PHP to the culture conditions increased cell size faster compared to normal culture under atmospheric pressure [123]. These findings suggested that supraphysiological PHP effectively induced cytoskeleton formation and ECM organization, including fibronectin fibrillogenesis, within a short period [18].

**Figure 3:**
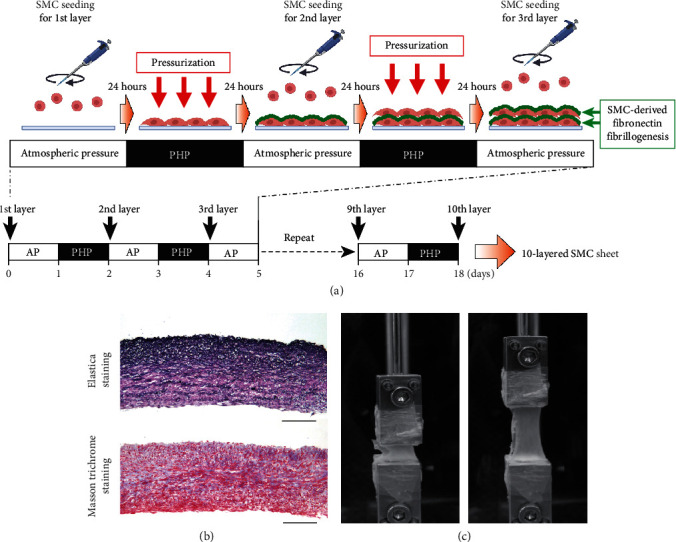
Fabrication procedure for SMC sheet containing layered elastic fibers. (a) Vascular SMCs were seeded on a cell culture disk to make the first SMC layer. Beginning 24 hours after seeding, SMCs were exposed to periodic hydrostatic pressure (PHP) for 24 hours. This PHP promoted SMC-derived fibronectin fibrillogenesis (illustrated as green color), which enabled the SMCs of the second layer to attach to the first layer. This procedure was repeated up to ten times to form a ten-layered SMC sheet. AP: atmospheric pressure. (b) A section of our ten-layered SMC sheet was stained with the Elastica van Gieson stain and the Masson trichrome stain to reveal the elastic fibers (deep purple color, upper panel) and collagens (blue color, lower panel), respectively. Scale bars: 100 *μ*m. (c) Our SMC sheet was able to be stretched with a DMT560 tissue puller (Danish Myo Technology, Aarhus N, Denmark). Stretched and unstretched SMC sheets are shown.

Rat neonatal aortic SMCs were seeded on a cell culture disk to create the first cell layer. Twenty-four hours after seeding, cells were exposed to PHP (110-180 kPa (65-590 mmHg), 0.002 Hz) for 24 hours, and then cells for the next layer were seeded on top of the first layer [18]. This procedure was repeated up to ten times. The result was the successful construction of a multilayered SMC sheet (Figure 3) [18]. This multilayered SMC sheet was readily detached from the cell culture disk and was then wrapped around a glass tube to form a tubular TEVG [18]. After two weeks of incubation with ascorbic acid, histological analysis revealed well-organized layered elastic fiber structures in this TEVG, comparable to those seen in the adult rat abdominal aorta [18]. The tensile rupture strength of these grafts was 1451 ± 159 mmHg [18], which is equivalent to the strength of the human saphenous vein [19]. In addition, this graft showed a linear pattern of stress-strain curves at low strain [18], which was similar to that seen in the rat aorta [18], suggesting that TEVGs generated according to this method possess functional elastic fibers. The fabricated TEVGs were able to be implanted in the rat aorta as patch grafts [18]. Doppler echocardiology showed no stenosis at 2.5 months after implantation [18]. Some capillary vessels were developed on the graft surface and abundant elastic fibers were retained [18], suggesting the biocompatibility of these grafts.

These studies have demonstrated the potential usefulness of supraphysiological hydrostatic pressurization in fabricating layered elastic fiber structures in cell-based TEVGs *in vitro* [18]. Emerging evidence suggests that hydrostatic pressure induces stress fiber formation in other cell types as well (e.g., MSCs [122], fibroblasts [124], and ECs [125]). Our novel approach may therefore be applicable to other cell types. Although further studies are required, this method may enable us to improve TEVGs such that their compliance will be comparable to that of native vessels, which will contribute to better clinical outcomes.

## 8. Conclusions

The field of TEVG generation continues to develop as clinical trials are conducted and novel technologies (e.g., iPSCs and 3D bioprinting) are reported. Several fabrication methods have been explored, yet the ideal method for TEVG production is yet to be achieved. One of the remaining issues is the lack of functional elastic fiber formation in TEVGs. Fortunately, our recent findings may further improve TEVG performance. Further integration of mechanics and biology should lead to novel findings and improved TEVG technologies, resulting in favorable outcomes for patients with cardiovascular diseases.
